# Parent‐reported compared with researcher‐measured child height and weight: impact on body mass index classification in Australian pre‐school aged children

**DOI:** 10.1002/hpja.702

**Published:** 2023-02-14

**Authors:** Jacklyn Kay Jackson, Alice Grady, Christophe Lecathelinais, Alison Fielding, Sze Lin Yoong

**Affiliations:** ^1^ Priority Research Centre for Health Behaviour College of Health, Medicine and Wellbeing, University of Newcastle Callaghan NSW Australia; ^2^ Hunter Medical Research Institute (HMRI) New Lambton Heights NSW Australia; ^3^ Hunter New England Population Health Unit, Hunter New England Local Health District Wallsend NSW Australia; ^4^ NSW & ACT Research and Evaluation Unit, GP Synergy, Regional Training Organisation (RTO) Mayfield West NSW Australia; ^5^ Global Centre for Preventive Health and Nutrition, Institute for Health Transformation, School of Health and Social Development, Deakin University Geelong Victoria Australia

**Keywords:** body height, body mass index, body weight, overweight, parents, preschool child, reliability, self‐report, validity

## Abstract

**Issue Addressed:**

Parent‐reported data may provide a practical and cheap way for estimating young children's weight status. This study aims to compare the validity and reliability of parent‐reported height and weight to researcher‐measured data for pre‐school aged children (aged 2‐6 years).

**Methods:**

This was a nested study within a cluster randomised controlled trial (October 2016‐April 2017), conducted within 32 Early Childhood Education and Care (ECEC) services across New South Wales, Australia. Parents of children reported on demographics and child height and weight via a survey. For the same child, height and weight data were objectively collected by trained research staff at the service. We calculated mean differences, intra‐class correlations, Bland‐Altman plots, percentage agreement and Cohen's kappa coefficient (>0.8 = “excellent”; 0.61‐0.8 = “good”; 0.41‐0.60 = “moderate”; 0.21 and 0.4 = “fair [weak]”; <0.2 = “poor”).

**Results:**

Overall, 89 children were included (mean age: 4.7 years; 59.5% female). The mean difference between parent‐reported and researcher‐measured data were small (BMI *z*‐score: mean difference −0.01 [95% CI: −0.45 to 0.44]). There was “fair/weak” agreement between parent‐categorised child BMI compared with researcher‐measured data (Cohen's Kappa 0.24 [95% CI: 0.06 to 0.42]). Agreement was poor (Cohen's kappa <0.2) for female children, when reported by fathers or by parents with a BMI > 25 kg/m^2^.

**Conclusion:**

There was “fair/weak” agreement between parent‐reported and measured estimates of child weight status.

**So What?:**

Parent's report of weight and height may be a weak indicator of adiposity at the level of individuals however it may be useful for aggregate estimates.

## 1 BACKGROUND

Childhood obesity is an excess in body fatness, frequently defined according to body mass index scores, adjusted for child sex and age. As childhood obesity can track throughout the lifespan^1^ and is associated with a higher risk of chronic diseases in childhood and later life^2^ early childhood is considered a critical period for directing public health obesity prevention efforts.^3^ In Australia, obesity has been a national health priority for over a decade, with the 2022 National Obesity Strategy providing a 10‐year framework to prevent and treat overweight and obesity.^4^, ^5^, ^6^

To support monitoring and understanding of the impact of public health interventions and policies, pragmatic and valid measures of child height and weight are needed to provide population estimates. While objectively measured height and weight, collected by trained personnel is the gold standard for determining BMI, this approach is often not feasible at a population level. As such, many studies utilise parent reported weight and height to assess child BMI, as it is a simple and cost‐effective method for collecting this information.^7^ Previous research internationally with pre‐school aged children found that carers could reasonably estimate children's weight and height however over 75% of obese children would be missed using parent self‐report.^8^, ^9^ To our knowledge, there have been no studies that have assessed this specifically in Australian preschool‐aged children (aged <6 years).

Therefore, the aim of this study was to, in children aged between 2 and 6 years old, (i) compare parent reported to researcher measured child height and weight; and (ii) explore validity of parent‐reported child height and weight classifications by child sex, or parent characteristics.

## 2 METHODS

### 2.1 Data source

This is a nested study using baseline data (collected between October 2016 and April 2017) from a cluster randomised controlled trial that aimed to investigate the impact of a web‐based menu‐planning intervention in Early Childhood Education and Care (ECEC) services. A protocol and outcome papers have been previously published outlining the methods and data collection in detail.^10^, ^11^, ^12^ Ethics approval was obtained from Hunter New England (16/02/17/4.05) and the University of Newcastle (H‐2016‐0111) Human Research Ethics Committees.

### 2.2 Participants

**FIGURE 1:**
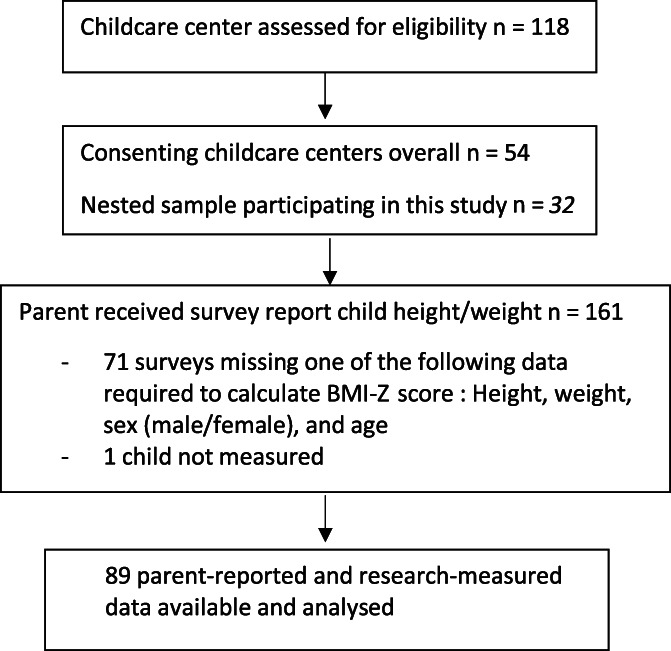
Participant flow diagram

Initially, eligible ECEC services were sent a recruitment package consisting of an invitation letter and information statement.^10^, ^11^, ^12^ A total of 54 services consented to larger trial and a nested sample of parents and children attending 35 ECEC services in New South Wales (NSW), Australia participated in this study (see Figure 1). All participating ECEC services distributed information and consent forms to parents of children in the room with the highest number of children aged 2‐6 years at baseline as a range of other data was also collected on the day. Consistent with previous approaches employed by the research team,^13^, ^14^ research assistants also approached parents at drop‐off to provide additional information about the study and enable return of consent forms on the day to increase participation. Eligible children were: (i) aged 2‐6 years; (ii) present at the centre on days of data collection; (iii) had no dietary requirements preventing consumption of foods while in care; and (iv) had parental consent.

### 2.3 Data collection

Consenting parents were invited to participate in an online or telephone survey. The survey collected: (i) parent demographic information including parent age, gender, height, weight, Aboriginal and Torres Strait Islander status, country of birth, language spoken at home, and highest education attainment and (ii) parent report of their child's height (without shoes, in cm/feet and inches) and weight (without clothes or shoes, in kg, pounds or ounces).

Objective measures of child height and weight were also collected from the same children on the day by trained research staff following a standardised protocol.^10^ Specifically, weight was measured using calibrated digital scales (NUWEIGH LOF842) and height was measured using a standing portable stadiometer (Charder HM 200P) on a hard, flat surface. Trained staff collected two measurements, from which the average of each measurement was used. The difference between measures was required to be 0.3 cm or less for height and 0.4 kg or less for weight, otherwise a third measure was taken. A third measurement for height was required in 13.5% of children, and none required a third measurement for weight.

For both parent‐report and researcher‐measured data, BMI was calculated as: weight (in kilograms) divided by height (in meters^2^). BMI *z*‐scores were calculated according to child age and sex and grouped according to cut‐offs defined by the World Health Organization.^15^

### 2.4 Statistical analysis

All data analysis was conducted using SAS software, version 9.3 (SAS Institute). Descriptive statistics, including means, frequencies and proportions were used to describe the demographic data. Parent and child data were only included if the child had both researcher‐measured and parent‐reported height and weight data. This resulted in data from 32 of the 35 participating services being included in the analysis.

For child height, weight, BMI (kg/m^2^), and BMI‐*z* scores differences between the two methods, we assessed mean estimates using linear mixed model regression controlling for clustering within ECC services. Subgroup analyses were conducted based on child sex (male vs female), and parent characteristics including parent sex (male vs female) and parent weight status (BMI ≤ 25 kg/m^2^ vs BMI > 25 kg/m^2^). Intra‐class correlations (ICC) were calculated and Bland‐Altman plots used to explore the agreement between individual's absolute values from each method.^16^, ^17^

To assess reliability related to parent‐reported BMI categorisation (Underweight; Healthy; Overweight/Obese), we calculated percentage agreement and weighted Cohen kappa coefficient (ranging from 0 to 1), were considered “excellent” if >0.8; “good (strong)” if between 0.61 and 0.8; “moderate” if between 0.41 and 0.60 agreement; “fair (weak to minimal)”^18^ if between 0.21 and 0.4; and “poor (almost none)” if values were less than 0.2.^19^

## 3 RESULTS

A total of 89 children from 32 ECEC services had both parent‐reported and researcher‐measured height and weight data available. Approximately 60% of ECEC services were located in high socioeconomic areas, 75% in major cities, and the number of children attending per day ranged from 21 to 87 children. Most parents were female (77.5%), with a mean age of 38 years and a high proportion of parents had obtained university level education (69%). The average age of participating children was 4.7 years, 59.5% were female and 23.9% of parents spoke a language other English at home (see Appendix A for characteristics of participating parents and children).

Overall, the mean difference between parent‐reported and researcher measured height, weight, BMI (kg/m^2^) and BMI *z* scores are small and not statistically significant (see Table 1). The lCC for was lowest for BMI (kg/m^2^) and BMI *z* scores (ICC = .16, indicating poor reliability), and highest for weight (ICC = .72, indicating moderate reliability). There were statistically significant differences in mean differences between measures for male child height (−2.00 [95% CI: −3.98 to −0.01] ICC = .18), BMI (1.28 [95% CI: 0.29 to 2.28] ICC = .07), BMI *z* sores (0.65 [95% CI: 0.09 to 1.20] ICC = .07). No other statistically significant differences were identified.

**TABLE 1:** Differences between parent reported and researcher measured child height and weight and BMI.

	Parent reported		Researcher measured		Mean difference^a^ (95% CI)	ICC
	Mean	SD	Mean	SD		
Total sample, N = 89						
Height (cm)	106.6	10.2	106.9	5.6	−0.27 (−2.16 to 1.6)	.32
Weight (kg)	18.2	3.0	18.3	2.9	−0.06 (−0.42 to 0.31)	.72
BMI (kg/m^2^)	16.3	3.8	15.9	1.5	0.40 (−0.38 to 1.19)	.16
BMI *z* score	0.36	2.3	0.37	0.95	−0.01 (−0.45 to 0.44)	.16
By child sex, female, N = 53						
Height (cm)	107.2	11.5	106.3	5.5	0.91 (−1.98 to 3.8)	.36
Weight (kg)	17.7	3.1	18.1	3.2	−0.41 (−0.90 to 0.07)	.49
BMI (kg/m^2^)	15.7	3.9	15.9	1.7	−0.19 (−1.32 to 0.93)	.03
BMI *z* score	−0.11	2.3	0.34	0.99	−0.45 (−1.08 to 0.18)	.14
By child sex, male, N = 36						
Height (cm)	105.8	7.8	107.8	5.8	−2.00 (−3.98 to −0.01)*	.18
Weight (kg)	18.9	2.7	18.5	2.4	0.47 (−0.05 to 0.98)	.34
BMI (kg/m^2^)	17.2	3.4	15.9	1.2	1.3 (0.29 to 2.28)*	.07
BMI *z* score	1.1	2.0	0.41	0.90	0.65 (0.09 to 1.2)*	.07
By parent gender, female, N = 69						
Height (cm)	107.4	9.9	107.2	5.6	0.25 (−2.02 to 2.5)	.35
Weight (kg)	18.3	3.0	18.4	2.9	−0.10 (−0.54 to 0.33)	.73
BMI (kg/m^2^)	16.1	3.4	15.9	1.5	0.16 (−0.77 to 1.1)	.26
BMI *z* score	0.25	2.2	0.37	0.94	−0.13 (−0.66 to 0.41)	.17
By parent gender, male, N = 20						
Height (cm)	103.9	10.8	105.9	5.8	−2.0 (−6.25 to 2.17)	.35
Weight (kg)	18.0	3.0	17.9	2.6	0.11 (−0.70 to 0.91)	.73
BMI (kg/m^2^)	17.2	5.0	15.9	1.6	1.07 (−0.90 to 3.0)	.26
BMI *z* score	0.77	2.3	0.36	1.0	0.41 (−0.58 to 1.40)	.17
By parent BMI, ≤25 kg/m^2^, N = 49						
Height (cm)	106.5	6.9	107.1	4.9	−0.64 (−2.17 to 0.90)	.28
Weight (kg)	18.2	2.4	17.9	1.9	0.22 (−0.16 to 0.61)	.71
BMI (kg/m^2^)	16.2	2.7	15.7	1.2	0.52 (−0.16 to 1.2)	.06
BMI *z* score	0.44	1.60	0.22	0.86	0.22 (−0.16 to 0.60)	.13
By parent BMI, >25 kg/m^2^, N = 32						
Height (cm)	107.7	13.8	106.3	6.7	1.4 (−3.0 to 5.8)	.28
Weight (kg)	17.9	3.6	18.4	3.9	−0.47 (−1.23 to 0.29)	.71
BMI (kg/m^2^)	16.0	5.0	16.1	1.8	−0.11 (−1.90 to 1.7)	.06
BMI *z* score	−0.11	2.9	0.45	1.0	−0.57 (−1.57 to 0.44)	.13

^a^ Linear mixed models regression with random effect accounting for clustering.

* *P* < .05.

**FIGURE 2:**
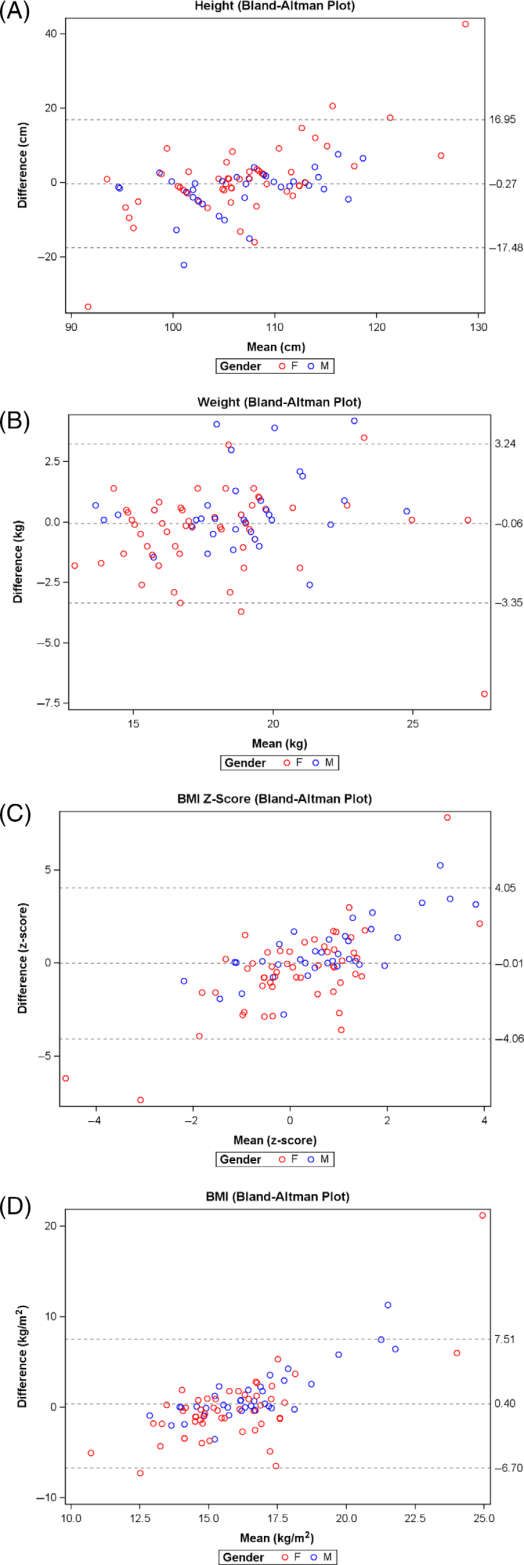
Bland‐Altman plots showing agreement between height, weight and BMI‐*z* scores, by researcher‐measured and parent‐reported data. (A) Height, (B) weight, (C) BMI‐*Z*‐score, (D) raw BMI (kg/m^2^). For each measurement, the mean difference between researcher measured and parent reported data (*y* axis) was plotted against the mean measurement (*x* axis), including mean differences and 95% CIs. The middle line represents the mean difference of the methods. Lines above and below represent 95% limits of agreement (LOA), where upper LOA is +1.96 SD and lower is −1.96 SD from overall mean differences

A visual examination of Bland‐Altman plots (Figure 2) indicated that, parents tend to overestimate if child height was above 110 cm, but underestimate if child height was below 110 cm (regression estimate: 0.7 [95% CI: 0.5 to 1.0; *P* < .0001]). The accuracy of parent reported weight varied regardless of child weight (regression estimate: 0.05 [95% CI: −0.08 to 0.2; *P* < .5]) (Figure 2A,B). This variation led to an overestimation in BMI ranging from 20 kg/m^2^ to underestimation of 7 kg/m^2^ (Figure 2D) (regression estimate: 1.2 [95% CI: 1.0 to 1.4; *P* < .0001]).

Percentage agreement between parent‐reported and research‐measured data on BMI classifications was 58%. Subgroup analyses revealed that percentage agreement between the measures was better for male children (67%); when reported by mothers (64%); and parents with a healthy BMI (≤25 kg/m^2^) (67%). Cohen's Kappa indicated fair agreement between the two measures overall (0.24 [95% CI: 0.06 to 0.42]), with “poor” agreement for female children (0.11 [95% CI: −0.12 to 0.35]), data reported by fathers (0.09 [95% CI: −0.24 to 0.41]), or by parents with a higher BMI (0.03 [95% CI: −0.25 to 0.31]) (see Table 2).

**TABLE 2:** Differences in body mass index classification using parent‐reported and researcher‐measured height and weight data.

BMI using parent‐reported data	BMI using researcher‐measured data					
	Obese/Overweight	Healthy weight	N exact match	Proportion exact match	Cohen's Kappa^a^ (95% CI)	*P*‐value symmetry test
Overall, N = 89						
Obese/Overweight	13	19	13	58%	0.24 (0.06 to 0.42)	.005
Healthy weight	9	39	39			
Underweight	0	9	‐			
By child sex, female, N = 53						
Obese/Overweight	5	11	5	53%	0.11 (−0.12 to 0.35)	.05
Healthy weight	7	23	23			
Underweight	0	7	‐			
By child sex, male, N = 36						
Obese/Overweight	8	8	8	67%	0.39 (0.12 to 0.66)	.13
Healthy weight	2	16	16			
Underweight	0	2	‐			
By parent gender, female, N = 69						
Obese/Overweight	10	12	10	64%	0.29 (0.08 to 0.51)	.06
Healthy weight	7	34	34			
Underweight	0	6	‐			
By parent gender, male, N = 20						
Obese/Overweight	3	7	3	40%	0.09 (−0.24 to 0.41)	.12
Healthy weight	2	5	5			
Underweight	0	3	‐			
By parent BMI, ≤25 kg/m^2^, N = 49						
Obese/Overweight	6	9	6	67%	0.29 (0.02 to 0.56)	.18
Healthy weight	4	27	27			
Underweight	0	3	‐			
By parent BMI, >25 kg/m^2^, N = 32						
Obese/Overweight	3	8	3	41%	0.03 (−0.25 to 0.31)	.08
Healthy weight	5	10	10			
Underweight	0	6	‐			

^a^ Weighted Cohen's Kappa.

## 4 DISCUSSION

Our study found that parents of Australian preschool‐aged children report on average only slightly below researcher‐measured height (mean difference: −0.27 cm) and weight (mean difference: −0.06 kg) data, which had minimal implications on calculated BMI *z* scores (mean difference: −0.01) and BMI (mean difference: 0.40). In terms of classification of weight status, parent‐reported data provided a fair (weak) estimate of child weight status categorisation (Cohen's Kappa: 0.24).

These results are consistent with international studies in preschool‐aged children which found that parents underestimate child BMI by between 0.3 and 0.5 kg/m^2^.^8^, ^9^ However, the agreement in BMI categorisation was weaker than a previous Australian study of children aged 4‐11 years which found moderate agreement for BMI categorisation (Cohen's kappa of 0.59) despite a larger underreporting of weight (0.5 kg) and height (0.9 cm) on average.^20^ Visual inspections of the plot also identified groupings of data values. This could be partly due to differences in the way weight and height were recorded. This was up to two decimal points for measured and usually in integer values for parents.

Self‐reported height and weight data represents a cost effective and pragmatic method for informing population health status at scale.^21^ It has been suggested that providing parents with explicit instructions when reporting anthropometric data could improve the accuracy of parent‐reported data. A range of reporting biases may impact the validity of self‐reported height and weight data; therefore, adjustment methods are commonly used in analysis models to improve self‐reported estimates. This study provides indication of differences in reporting with better estimates for BMI categorisation when the child was male, where reported by mothers, and parents were ≤25 kg/m^2^. Such findings are consistent with previous studies internationally.^8^ A study with over 9000 German children found that girls were more likely to be misclassified then boys, and parent's perception of weight status (girls being perceived as “too fat” and boy perceived as “too thin”) influenced this report.^22^ It is perhaps unsurprising that mothers report more accurately on their children's weight and height, given they commonly hold the role of primary carer.

The primary limitations of our current investigation are a relatively small sample of participants, attending ECEC services recruited to participate in a web‐based menu‐planning program, relatively low service response rate (47%) and cross‐sectional study design. The findings are however consistent with the only other Australian study with younger children.^20^

## 5 CONCLUSIONS

In a sample of Australian pre‐school aged children,  we found little difference in mean BMI *z*‐scores between parent‐reported and research measured data. Despite this, reliability of BMI categorisation was weak suggesting that weight and height data could provide reasonable estimates for population or large‐scale studies, however this is unlikely to be an accurate indicator of adiposity at the individual level. Further exploration of the factors that influence parent‐report is needed in more representative samples of Australian pre‐school aged children.

## FUNDING INFORMATION

This project was funded by the National Health and Medical Research Council (NHMRC) (APP1102943), Cancer Council NSW (PG 16‐05), and the Priority Research Centre for Health Behaviour, University of Newcastle. The content of this publication is the responsibility of the authors and do not reflect views of funding bodies. Alice Grady receives salary support from the Heart Foundation Postdoctoral Fellowship (102518). Sze Lin Yoong was supported by a Heart Foundation Postdoctoral Fellowship at the time of the research (ref no:100547) from the National Heart Foundation of Australia.

## CONFLICT OF INTEREST STATEMENT

The authors declare no conflict of interest.

## ETHICS STATEMENT

Ethics approval was obtained from Hunter New England (16/02/17/4.05) and the University of Newcastle (H‐2016‐0111) Human Research Ethics Committees.

## Data Availability

The data that support the findings of this study are available from the corresponding author upon reasonable request.

## APPENDIX A

Demographic characteristics of participating parents and their children (N = 89), as reported by parents.

**Table:**

Characteristic	N or mean	% or SD
Parents		
Age (years), mean (SD)	37.6	6.2
Male sex, N (%)	20	22.5
Female sex, N (%)	69	77.5
Aboriginal or Torres Strait Islander status, N (%)	1	1.1
Language other than English spoken at home^a^, N (%)	21	23.9
BMI weight category (self‐reported)		
Underweight, N (%)	1	1.23
Healthy weight, N (%)	48	59.3
Overweight, N (%)	21	25.9
Obese, N (%)	11	13.6
Education level		
School certificate, N (%)	6	6.7
Higher school certificate, N (%)	10	11.2
Certificate or diploma, N (%)	12	13.5
University or other tertiary institute degree, N (%)	61	68.5
Country of birth^a^		
Australia, N (%)	57	64.8
Other, N (%)	31	35.2
Children		
Age (years), mean (SD)	4.7	0.6
Male sex, N (%)	36	40.5
Female sex, N (%)	53	59.6

^a^ N = 88.

## References

[1] Ward ZJ , Long MW , Resch SC , Giles CM , Cradock AL , Gortmaker SL . Simulation of growth trajectories of childhood obesity into adulthood. N Engl J Med. 2017;377:2145–53. 29171811 10.1056/NEJMoa1703860 PMC9036858

[2] Umer A , Kelley GA , Cottrell LE , Giacobbi P Jr , Innes KE , Lilly CL . Childhood obesity and adult cardiovascular disease risk factors: a systematic review with meta‐analysis. BMC Public Health. 2017;17(1):683. 28851330 10.1186/s12889-017-4691-z PMC5575877

[3] World Health Organization . Commission on ending childhood obesity. Facts and figures on childhood obesity. 2017. Available from: https://www.who.int/end-childhood-obesity/facts/en/.

[4] Rissel C , Innes‐Hughes CJ , Thomas M , Wolfenden L . Reflections on the NSW Healthy Children Initiative: a comprehensive state‐delivered childhood obesity prevention initiative. Public Health Res Pract. 2019;29(1):2911908. 30972409 10.17061/phrp2911908

[5] Hayes A , Tan EJ , Lung T , Brown V , Moodie M , Baur L . A new model for evaluation of interventions to prevent obesity in early childhood. Front Endocrinol (Lausanne). 2019;10:132. 30881347 10.3389/fendo.2019.00132 PMC6405882

[6] Commonwealth of Australia . The National Obesity Strategy 2022‐2032. Health Ministers Meeting. 2022.

[7] Australian Bureau of Statistics . National Health Survey: First results: Australian Bureau of Statistics; 2018. Available from: https://www.abs.gov.au/statistics/health/health‐conditions‐and‐risks/national‐health‐survey‐first‐results/latest‐release#about‐the‐national‐health‐survey.

[8] Dubois L , Girad M . Accuracy of maternal reports of pre‐schoolers' weights and heights as estimates of BMI values. Int J Epidemiol. 2007;36(1):132–8. 17510077 10.1093/ije/dyl281

[9] Huybrechts I , Himes JH , Ottevaere C , De Vriendt T , De Keyzer W , Cox B , et al. Validity of parent‐reported weight and height of preschool children measured at home or estimated without home measurement: a validation study. BMC Pediatr. 2011;11:63. 21736757 10.1186/1471-2431-11-63 PMC3149571

[10] Yoong SL , Grady A , Wiggers J , Flood V , Rissel C , Finch M , et al. A randomised controlled trial of an online menu planning intervention to improve childcare service adherence to dietary guidelines: a study protocol. BMJ Open. 2017;7(9):e017498. 10.1136/bmjopen-2017-017498 PMC5595182 28893755

[11] Grady A , Wolfenden L , Wiggers J , Rissel C , Finch M , Flood V , et al. Effectiveness of a web‐based menu‐planning intervention to improve childcare service compliance with dietary guidelines: randomized controlled trial. J Med Internet Res. 2020;22(2):e13401. 32014843 10.2196/13401 PMC7055768

[12] Yoong SL , Grady A , Wiggers JH , Stacey FG , Rissel C , Flood V , et al. Child‐level evaluation of a web‐based intervention to improve dietary guideline implementation in childcare centers: a cluster‐randomized controlled trial. Am J Clin Nutr. 2020;111(4):854–63. 32091593 10.1093/ajcn/nqaa025 PMC7138676

[13] Yoong SL , Grady A , Stacey F , Polimeni M , Clayton O , Jones J , et al. A pilot randomized controlled trial examining the impact of a sleep intervention targeting home routines on young children's (3‐6 years) physical activity. Pediatr Obes. 2019;14(4):e12481. 30417593 10.1111/ijpo.12481

[14] Wolfenden L , Jones J , Parmenter B , Razak LA , Wiggers J , Morgan PJ , et al. Efficacy of a free‐play intervention to increase physical activity during childcare: a randomized controlled trial. Health Educ Res. 2019;34(1):84–97. 30445644 10.1093/her/cyy041

[15] World Health Organization . WHO child growth standards: length/height‐for‐age, weight‐for‐age, weight‐for‐length, weight‐for‐height and body mass index‐for‐age: methods and development. Geneva: World Health Organization; 2006.

[16] Bland JM , Altman DG . Measuring agreement in method comparison studies. Stat Methods Med Res. 1999;8(2):135–60. 10501650 10.1177/096228029900800204

[17] Landis JR , Koch GG . The measurement of observer agreement for categorical data. Biometrics. 1977;33(1):159–74. 843571

[18] Fleiss JL , Cohen J . The equivalence of weighted kappa and the intraclass correlation coefficient as measures of reliability. Educ Psychol Meas. 1973;33(3):613–9.

[19] Watson PF , Petrie A . Method agreement analysis: a review of correct methodology. Theriogenology. 2010;73(9):1167–79. 20138353 10.1016/j.theriogenology.2010.01.003

[20] Chai LK , Collins CE , May C , Holder C , Burrows TL . Accuracy of parent‐reported child height and weight and calculated body mass index compared with objectively measured anthropometrics: secondary analysis of a randomized controlled trial. J Med Internet Res. 2019;21(9):e12532. 31538954 10.2196/12532 PMC6754693

[21] Davies A , Wellard‐Cole L , Rangan A , Allman‐Farinelli M . Validity of self‐reported weight and height for BMI classification: a cross‐sectional study among young adults. Nutrition. 2020;71:110622. 31837644 10.1016/j.nut.2019.110622

[22] Brettschneider AK , Ellert U , Schaffrath RA . Comparison of BMI derived from parent‐reported height and weight with measured values: results from the German KiGGS study. Int J Environ Res Public Health. 2012;9(2):632–47. 22470314 10.3390/ijerph9020632 PMC3315268

